# Risky Driving Behavior Recognition Based on Vehicle Trajectory

**DOI:** 10.3390/ijerph182312373

**Published:** 2021-11-24

**Authors:** Shengdi Chen, Qingwen Xue, Xiaochen Zhao, Yingying Xing, Jian John Lu

**Affiliations:** 1 Shandong Provincial Key Laboratory of Highway Technology and Safety Assessment, Shandong 250357, China; sdchen@shmtu.edu.cn; 2 College of Transport & Communications, Shanghai Maritime University, Shanghai 201306, China; 3 The Key Laboratory of Road and Traffic Engineering, Ministry of Education, College of Transportation Engineering, Tongji University, Shanghai 201804, China; zhao_xiaochen@tongji.edu.cn (X.Z.); yingying199004@163.com (Y.X.); jianjohnlu@tongji.edu.cn (J.J.L.)

**Keywords:** traffic safety, risky driving behavior recognition, vehicle trajectory, MOR, threshold value

## Abstract

This paper proposes a measurement of risk (MOR) method to recognize risky driving behavior based on the trajectory data extracted from surveillance videos. Three types of risky driving behavior are studied in this paper, i.e., speed-unstable driving, serpentine driving, and risky car-following driving. The risky driving behavior recognition model contains an MOR-based risk evaluation model and an MOR threshold selection method. An MOR-based risk evaluation model is established for three types of risky driving behavior based on driving features to quantify collision risk. Then, we propose two methods, i.e., the distribution-based method and the boxplot-based method, to determine the threshold value of the MOR to recognize risky driving behavior. Finally, the trajectory data extracted from UAV videos are used to validate the proposed model. The impact of vehicle types is also taken into consideration in the model. The results show that there are significant differences between threshold values for cars and heavy trucks when performing speed-unstable driving and risky car-following driving. In addition, the difference between the proportion of recognized risky driving behavior in the testing dataset compared with that in the training dataset is limited to less than 3.5%. The recognition accuracy of risky driving behavior with the boxplot- and distribution-based methods are, respectively, 91% and 86%, indicating the validation of the proposed model. The proposed model can be widely applied to risky driving behavior recognition in video-based surveillance systems.

## 1. Introduction

Driving safety is influenced by different factors (e.g., drivers, traffic environment, vehicle types), with the driver being one of the most important. It has been shown that about 95% of traffic accidents in China are caused by drivers [1]. Risky driving behavior of drivers results in crashes on the road [2]. Risky driving behavior refers to the unsafe or illegal driving behavior of drivers to realize the driving intention, such as arriving at the destination as soon as possible. The accurate and timely recognition of risky driving behavior will prevent traffic accidents and improve traffic safety [2].

There are several classification methods of risky driving behavior [3,4], which are summarized in Table 1. Kaufman et al. [3] classified risky driving behavior into aggressive driving and assertive driving based on drivers’ psychology. Driving skills are also classified into different groups, such as skilled safe driving, aggressive driving, unskilled driving, and conservative driving [5]. Some studies classified risky driving behavior based on traffic flow characteristics and occurrence frequency under different environments [6]. Li [7] classified risky driving behavior under snow and ice conditions into four types, i.e., overspeed driving, near car-following, illegal overtaking, and driving on the central lines by analyzing the features of roads and the environment. Si [8] studied risky driving behavior on highways such as fatigue driving, overspeed driving, illegal overtaking, frequent lane changing, driving on curves without slowing down, etc. Commercial vehicles have also been studied based on driving features [9]. In addition, risky driving behavior is also classified into different types based on crash severity such as major accidents, minor, or general accidents [10].

In recent years, there have been a lot of studies to recognize risky driving behavior and evaluate driving style [3,11]. Various data collection methods exist, such as naturalistic driving experiments, vehicle-based sensors, and driving simulation. With naturalistic driving experiments, researchers installed sensors and equipment on vehicles to collect operation data, driving data, and environment data [3]. Although high-precision vehicle motion data can be collected with naturalistic driving experiments, the cost of equipment is high, and vehicle-equipped sensors (e.g., cameras, smartphones) may affect the driving behavior of drivers, thus leading to abnormal driving behavior [12]. Smartphones have gradually been applied to collect trajectory data [13,14,15]. However, compared with cameras, such as surveillance video, smartphones can only collect the data of subject vehicles rather than the relative relationships between subject and adjacent vehicles. Therefore, it is difficult to evaluate the interaction between two vehicles. Additionally, smartphones cause privacy problems for drivers. For example, the specific position of vehicles will be located, which is private to drivers. Some studies used vehicle-based sensors such as gyroscopes [16] and accelerometers [17] to extract longitudinal and lateral speed and acceleration as data sources to analyze driving behavior. Although the cost of vehicle-based sensor data remains low, it limits the data type [18]. For example, accelerometers can only collect acceleration data of vehicles, and they have no access to the relative distance or speed between subject and preceding vehicles. Driving simulation experiments are also important data sources [19,20]. Although driving simulation experiments can simulate driving behavior under extreme conditions, they depend on the reliability of the driving environment design.

Compared with the above data collection methods, video surveillance systems have obvious advantages. Road surveillance systems have been widely developed in China, and they can collect more vehicle trajectory data and surrounding traffic environment data at the same time. In addition, video surveillance systems can capture the naturalistic driving of drivers without causing a disturbance to drivers. Trajectory data extracted from video surveillance systems have been widely applied in risky driving behavior recognition research [21,22,23]. However, in order to establish a recognition model, risky driving behavior needs to be labeled to provide training data, which would require experts to analyze video, resulting in inefficiency [22].

The threshold method is one of the most commonly used recognition methods for risky driving behavior recognition. Dingus et al. [24] proposed a threshold set of vehicle kinematics parameters, including lateral acceleration, longitudinal acceleration, and yaw rate based on a naturalistic driving dataset. A possible collision event would be labeled if any of a vehicle’s kinematics parameters exceeded the threshold value. Malta et al. [25] proposed a new threshold set of kinematics parameters based on the test data of a European active safety system. Based on basic acceleration and other parameters, headway, lane change time, and other parameters were added to the threshold set. Cheol et al. [26] determined the recognition threshold through the parameters of vehicle position, speed, acceleration, and angular velocity of risky driving events (e.g., sudden acceleration and deceleration and sudden lane change events) in a training dataset to detect risky driving behavior. Fitch et al. [27] considered road type when determining the threshold of the identification parameters of risky driving behavior. For example, when a vehicle travels at a speed of 64 km/h on a highway, it is marked as risky driving behavior when the longitudinal acceleration exceeds −0.3 g. However, most threshold methods do not consider changeable traffic environment conditions, and the proposed threshold exhibits good performance in similar datasets but may not be applicable to other environments. In addition, commonly used vehicle kinematics parameters (e.g., speed, acceleration) are often used as feature indicators.

In this paper, a risky driving behavior recognition model is proposed based on the trajectory data extracted from videos. The model contains two parts: an MOR-based risk evaluation model and an MOR threshold selection method. The MOR-based risk evaluation method establishes the MOR formulation for three types of risky driving behavior, i.e., speed-unstable driving, serpentine driving, and risky car-following driving. The driving features of risky driving behavior are extracted as parameters to establish the MOR formulation. Then, the threshold of the MOR is selected based on the distribution-based method and the boxplot-based method to recognize risky driving behavior. Finally, the risky driving behavior recognition mode is verified based on the trajectory data. The research results can be applied to the real-time detection of risky driving behavior in video surveillance systems and provide support for accidents prevention and traffic management.

## 2. Risky Driving Behavior Recognition Model

A risky driving behavior recognition model is established in this paper to quantify collision risk based on driving features and risk measurements. There are two parts to the model:

(1) MOR-based risk evaluation method. The MOR is proposed in terms of the driving features of risky driving behavior. The MOR can evaluate the risk of driving behavior in real time based on driving trajectory data.
(2) MOR threshold selection method. The distribution-based method and boxplot-based method are adopted to determine the threshold of the MOR based on trajectory data.

**Figure 1:**
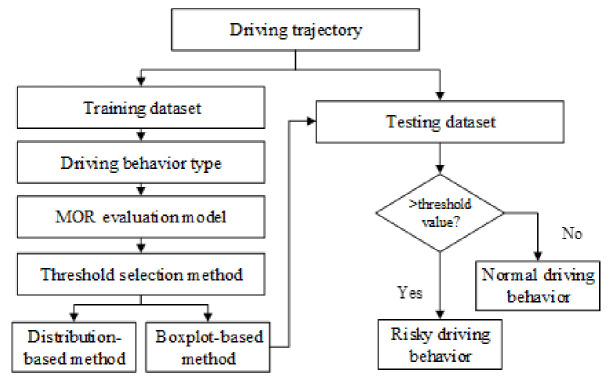
Process of the risky driving behavior recognition model.

Then, the threshold of the MOR is verified based on the testing data to recognize risky driving behavior. The process of the risky driving behavior recognition model is shown in Figure 1.

### 2.1. MOR-Based Risk Evaluation Method

Driving behavior can be classified into lane-keeping and lane-changing maneuvers when driving on the road. As for risky lane-keeping maneuvers, we mainly study speed-unstable driving, serpentine driving, and risky car-following driving. As there are not enough lane-changing samples extracted from the videos, we do not study risky lane-changing behavior in this paper. According to the characteristics of driving behavior, we establish the MOR to recognize risky driving behavior with easily accessible variables from videos. The MORs are as below.

(1) Speed-unstable driving

Speed-unstable driving is when a vehicle frequently accelerates or decelerates during the driving process. It can result in the misjudgment of a preceding vehicle’s movement for the following vehicles, thus increasing the rear-end crash probability. In order to reflect the speed fluctuation and variability during the driving process, we select the coefficient of variation [28] as MOR_1_ to indicate the risk of speed-unstable driving.

(1) $$MOR_{1}=\frac{SD(v)}{Mean(v)}\times 100\%$$

where SD(*v*) is the standard deviation of the speed, and mean(*v*) is the mean value of the speed. The driving speed stays more stable with a smaller value of MOR_1_.

(2) Serpentine driving

Serpentine driving is when a vehicle frequently swings from one side of the road to the other, presenting a serpentine driving state. It is easy to disturb surrounding drivers’ sight with frequent lateral swinging. It can make surrounding drivers unaware of the accurate traffic environment and unable to respond to the abrupt deceleration or turning of other vehicles, resulting in traffic crashes.

The lateral swing distance from one time step to the next can reflect the swing severity of serpentine driving; therefore, it is adopted as a feature to establish the MOR for serpentine driving. MOR_2_ is defined as the cumulative distance of lateral swing during a certain period, as shown in Equation (2).

(2) $$MOR_{2}=\sum_{t=2}^{N}\left|y(t)-y(t-1)\right|$$

where *y*(*t*) is the lateral position of the vehicle at time step *t*, and *y*(*t*−1) is the lateral position of the vehicle at time step *t−*1. A smaller value of MOR_2_ indicates a stable driving trajectory.

(3) Risky car-following

The car-following (CF) maneuver describes the interactive relationship between two following vehicles. However, the following vehicle will not be influenced by the preceding vehicle if there is a large relative distance between the two vehicles. According to Zhu et al. [29], a CF period was ultimately extracted if the following criteria were met simultaneously: (1) a leading vehicle exists; (2) gap < 120 m (this criterion eliminated free-flow traffic conditions); (3) duration of following period > 15 s (this criterion guaranteed that the CF persisted long enough to be analyzed). The CF samples are extracted from the CF period, whose time interval is defined as 4 s.

Risky CF maneuvers are mainly caused by the shorter relative distance and higher velocity of the following vehicles, resulting in a rear-end crash, as there is not enough time for the following vehicle to take counter maneuvers while the preceding vehicle abruptly decelerates. There have been some risk surrogates to describe the rear-end crash risk, e.g., time to collision (TTC) [30,31], modified time to collision (MTTC) [32], and time to collision with disturbance (TTCD) [33]. The TTC has been adopted as the standard collision warning parameter for vehicle collision avoidance systems or driver assistance systems. However, it cannot describe the collision risk when the relative velocity of two following vehicles is 0. Therefore, the inverted TTC (ITTC) is adopted in this paper as the MOR to evaluate the rear-end collision risk of CF maneuvers, as shown in Equation (3).

(3) $$MOR_{3}=\frac{v_{i-1}(t)-v_{i}(t)}{x_{i-i}(t)-x_{i}(t)-l_{i-1}}$$

where *v_i−1_*(*t*) is the speed of the following vehicle *i−*1 at time step *t*, *v_i_*(*t*) is the speed of the preceding vehicle *i* behind at time step *t*, *x_i−1_*(t)is the end position of the following vehicle *i−*1, *x_i_*(*t*)is the end position of the preceding vehicle *i*, and *l_i−_*_1_ is the length of the following vehicle *i−*1. The collision risk of car-following maneuvers is lower with smaller values of MOR_3_.

### 2.2. MOR Threshold Selection Method

The threshold value of the MOR needs to be determined as a criterion to classify risky driving behavior. In particular, the threshold value is influenced by the road, traffic environment, individuals, and vehicles. For example, the same driving trajectory at free flow and congested flow would result in different levels of collision risk. Therefore, the threshold value is not a specific value for all traffic environments. We can apply the threshold selection method to different traffic environments to analyze driving data and obtain the corresponding threshold value. In this paper, we adopt two methods, i.e., the boxplot-based method and the distribution-based method, to determine the threshold of the MOR based on training data.

#### 2.2.1. Boxplot-Based Method

**Figure 2:**
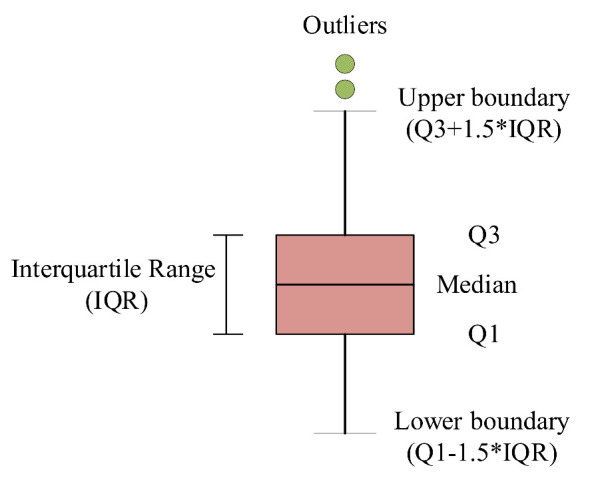
Box-and-whisker plot.

Risky driving behavior is usually the abnormal trajectory data to a normal driving trajectory, whose MOR values are extraordinarily higher compared with the values of normal driving behavior. The boxplot method is useful for recognizing abnormal points (i.e., outliers), as shown in Figure 2. Boxplots visually show the distribution of numerical data and skewness by displaying data quartiles (or percentiles) and averages. Box plots show the five-number summary of a set of data, including the upper boundary, first quartile, median, third quartile, and lower boundary. As shown in Figure 2, Q1 and Q3 are, respectively, the first quartile and third quartile of the data. Then, the interquartile range (IQR) can be obtained as the difference between Q1 and Q3. The upper boundary and lower boundary of the MOR boxplot can be determined with the IQR, Q3, and Q1. The 1.5 coefficient is the most selected value in related research to detect outliers [34]. The outliers beyond the upper boundary or below the lower boundary are the abnormal data, i.e., risky driving behavior. Risky driving behavior can be recognized with the boxplot method.

#### 2.2.2. Distribution-Based Method

Some researchers also adopted the statistical method to determine threshold values. For example, the 85th percentile of speed is normally assumed to be the highest safe speed for a roadway section [35]. Therefore, the distribution-based method is also applied in this paper to select the threshold value of the MOR. Videos can capture mass driving trajectory data under similar environments within a limited period, providing enough training data to use in the distribution-based method.

The process of the distribution-based method is detailed as below:

(1) Different types of risky driving behavior samples are extracted from the trajectory dataset, and the samples are classified into the training set and testing set. Each type of risky driving behavior is included in the two sets.
(2) The MOR value of each driving behavior sample is calculated based on the MOR-based risk evaluation method.
(3) The cumulative distribution curve for all driving behavior samples of one type is obtained, and the percentile value is obtained as a threshold value based on the training dataset.
(4) The threshold value is used to recognize risky driving behavior in the test dataset.

## 3. Data Acquisition and Processing

**Figure 3:**
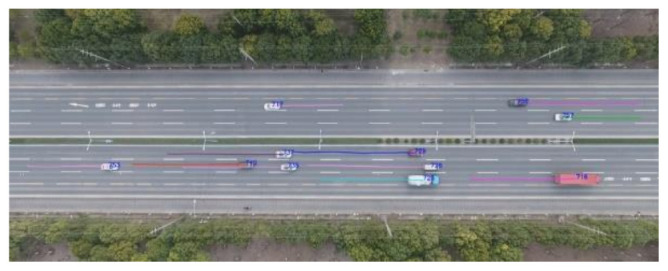
UAV video screenshot of road traffic and trajectory extraction.

The length of the UAV video coverage area is about 250 m. The time period of each video is about 15 min due to the battery. Thirty videos were collected at a highway in Shanghai, China, during off-peak hours from 10:00 a.m. to 12:00 a.m. The highway consists of eight lanes, including two right-turn lanes and two left-turn and straight lanes. Video processing software developed by Nanjing University of Science and Technology was used to extract the vehicle driving data from the UAV videos, as shown in Figure 3. The vehicle information that the software can directly extract includes vehicle ID, time, position information, speed, acceleration, vehicle type, and preceding vehicle ID. It can help to match two following vehicles, which can help to study the relative position and assess collision risk. The data extraction frequency was 10 Hz. The extracted vehicle behavior trajectory can be stored in Excel and imported into MATLAB software for analysis. In order to ensure the accuracy of data extraction, the difference between the actual speed of a naturalistic driving vehicle (measured directly by equipped sensors) and the extracted speed (UAV video) was within 3.5%.

This paper selected three maneuver types to study. Although current image recognition and machine learning technologies can ensure that the trajectory data extracted from videos have high accuracy, some errors are inevitable, which leads to some noises in the trajectory data. The data extraction and process are depicted as follows.

(1) The software extracts vehicle ID, lateral and longitudinal speed, acceleration, vehicle length and width, lane ID, position information, and preceding vehicle ID every 0.1 s.
(2) It deletes abnormal IDs that remain static and IDs that cannot be further matched and processed.
(3) For the missing frames in the extracted trajectory data, the cubic spline interpolation [36] method is used to fill in information such as position, velocity, and acceleration. Then, the sliding time window method is applied to eliminate abnormal data and noises.
(4) By matching the ID and time stamp between the preceding and following vehicles, the relative distance and velocity between two vehicles are calculated.

The data process can provide data for the risky driving behavior recognition model. We selected 600 vehicles from videos to study in this paper. The trajectory data of each vehicle were divided into different types of maneuvers based on the sample extraction standard mentioned above. The sample distribution is shown in Table 2.

## 4. Threshold Value Selection and Comparison

### 4.1. Threshold Value Based on Boxplot

The threshold value of the three types of risky driving behavior was calculated by the boxplot-based method using the training dataset. The results are analyzed as follows.

(1) Speed-unstable driving

**Figure 4:**
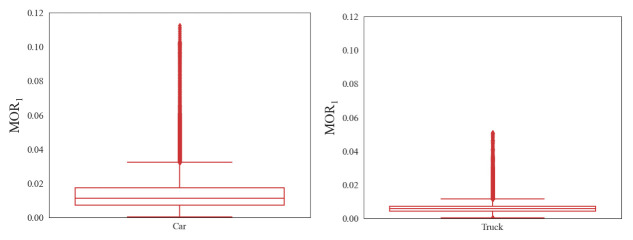
Box-and-whisker plot of MOR_1_.

A boxplot of speed-unstable driving samples is shown in Figure 4. It is known that a smaller value of MOR_1_ indicates lower collision risk. Therefore, the upper boundary was taken as the threshold value to detect risky driving behavior. The threshold values of MOR_1_ for cars and heavy trucks are, respectively, 0.03 and 0.01. The threshold value for heavy trucks is much lower than that of passenger cars, indicating that heavy truck drivers tend to maintain a stable driving velocity. This may result from the fact that the acceleration and deceleration performance for heavy trucks is worse than that of passenger cars.

(2) Serpentine driving

**Figure 5:**
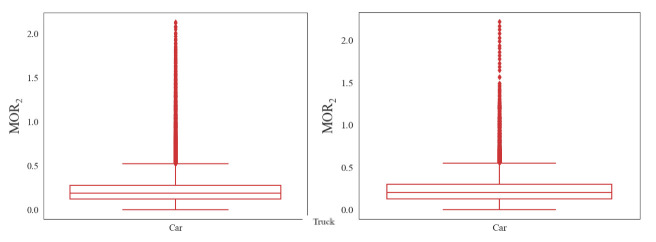
Box-and-whisker plot of MOR_2_.

A smaller MOR_2_ indicates less lateral swing of vehicles and less collision risk. Therefore, the upper boundary of the boxplot was selected as the threshold value to detect risky serpentine driving, as shown in Figure 5. The threshold values of MOR_2_ for cars and heavy trucks are, respectively, 0.54 m and 0.69 m. The threshold value for heavy trucks is higher than that of passenger cars.

(3) Risky car-following

**Figure 6:**
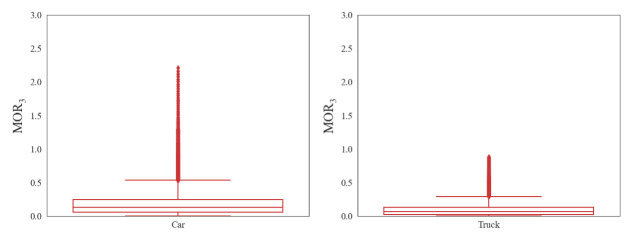
Box-and-whisker plot of MOR_3_.

A smaller value of MOR_3_ indicates less crash possibility to the preceding vehicle. It was investigated that there is no possibility to have a crash with a negative ITTC. To be more specific, the value of the ITTC is negative if the preceding vehicle’s speed is higher than that of the following vehicle. Then, it will not cause a crash. Therefore, we only considered the positive ITTC and extracted the upper outliner of the boxplot. As shown in Figure 6, the threshold values of MOR_3_ for passenger cars and heavy trucks are, respectively, 0.58 s^−1^ and 0.35 s^−1^. The threshold values for heavy trucks are much lower than those of passenger cars. This may be explained by the fact that the deceleration performance of heavy trucks is worse than that of cars, so truck drivers need to maintain a larger distance to preceding vehicles to avoid a collision.

### 4.2. Threshold Value Based on Distribution

The distribution of the MOR for each risky driving maneuver was fitted by an appropriate curve. In particular, the MOR distribution of cars and trucks was analyzed due to the performance difference. The distribution results are detailed as follows.

(1) Speed-unstable driving

**Figure 7:**
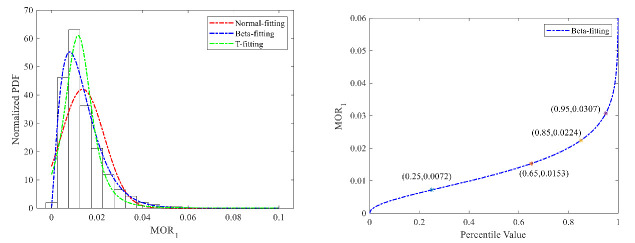
MOR1 fitting results and threshold for cars.

**Figure 8:**
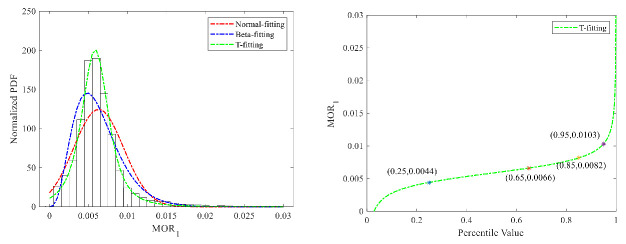
MOR_1_ fitting results and threshold for trucks.

We adopted the Normal distribution, Beta distribution, and T distribution to fit the MOR_1_ distribution, as shown in Figure 7. The mean-square error (MSE) between the distribution fitting curve and real data were calculated for the three types of distribution to select the best one. The MSE values for the three distributions are, respectively, 12.03, 10.98, and 11.05. According to the fitting results, the Beta distribution can better describe the features of the MOR_1_ distribution. Therefore, the Beta distribution was selected to fit the distribution. The slope of the cumulative distribution curve changed greatly at 90–99%. Therefore, we selected the 95% percentile value as the threshold value, which is 0.03 for car drivers. The fitting results of truck drivers are shown in Figure 8, and the T distribution shows better performance. The 95% percentile value of the T cumulative distribution curve was calculated as the threshold value, which is 0.01. The threshold value of heavy trucks is still lower than that of passenger cars.

(2) Serpentine driving

**Figure 9:**
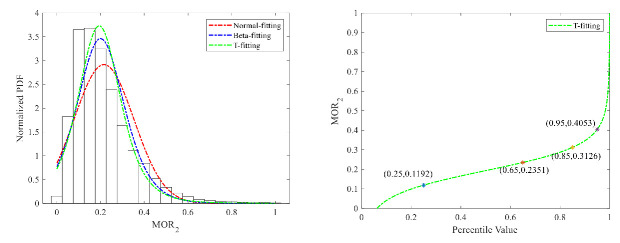
MOR_2_ fitting results and threshold for cars.

**Figure 10:**
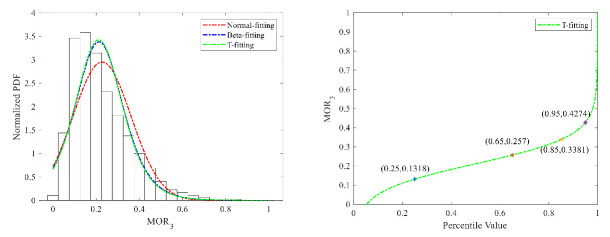
MOR_2_ fitting results and threshold for trucks.

The fitting results of the three distributions of MOR_2_ are shown in Figure 9 and Figure 10. Because of the good fitting performance of the T distribution, the 95% percentile value was selected as the threshold value to detect risky serpentine driving. The threshold values for cars and trucks are, respectively, 0.41 m and 0.43 m. The threshold value of heavy trucks is higher than that of cars.

(3) Risky car-following

**Figure 11:**
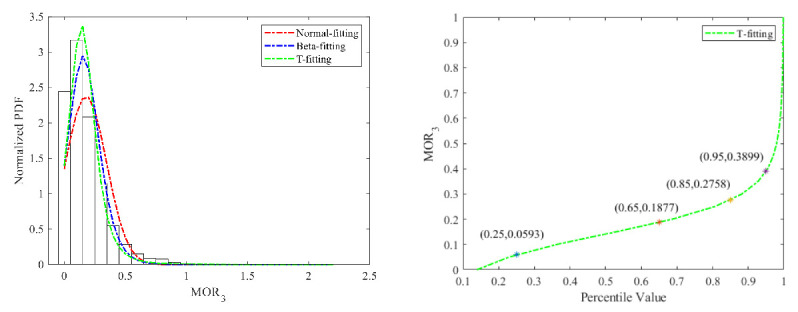
MOR_3_ fitting results and threshold for cars.

**Figure 12:**
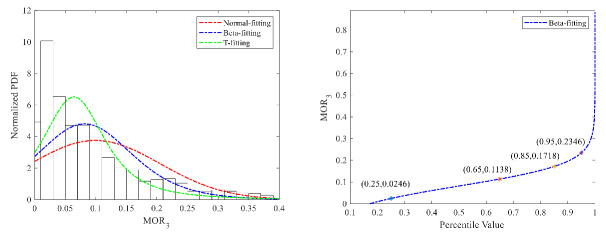
MOR_3_ fitting results and threshold for trucks.

The fitting results of the Normal distribution, Beta distribution, and T distribution are shown in Figure 11 and Figure 12. While the fitting performance of the Beta distribution was better, the 95% percentile value of the T cumulative probability curve was selected as the threshold value to recognize risky car-following maneuvers, which is 0.38 s^−1^ for car drivers. The Beta distribution was adopted for truck drivers, and the threshold value to recognize risky following maneuvers for truck drivers is 0.23 s^−^^1^.

### 4.3. Comparison and Analysis of Results

In order to validate the threshold value, we analyzed the proportion of risky driving maneuvers in the training dataset and testing dataset. To be more specific, the threshold value determined by the boxplot- or distribution-based method was applied to the testing set for the recognition of risky driving maneuvers. Then, the proportion of recognized risky maneuvers in the testing set was compared with that in the training dataset. The threshold value proved to be efficient if there was little difference between the two proportion values.

The proportion of risky driving maneuvers with the threshold value of the boxplot-based method is shown in Table 3. The absolute differences between the training dataset and testing dataset are 0.73% and 0.17% in the speed-unstable driving proportion for car and truck drivers, 0.02% and 3.35% in serpentine driving, and 0.57% and 1.39% in risky car-following. The average difference is 1.04%. The insignificant differences between the training and testing datasets in risky driving maneuvers recognition indicate the efficiency of the threshold value.

The proportion of risky driving maneuvers with the threshold value of the distribution-based method is shown in Table 4. The absolute differences between the training dataset and testing dataset are 1.77% and 0.38% in the speed-unstable driving proportion for car and truck driving, 0.57% and 2.67% in serpentine driving, and 1.93% and 1.68% in risky car-following driving. The average difference is 1.18%. This also indicates the efficiency of the threshold value.

**Figure 13:**
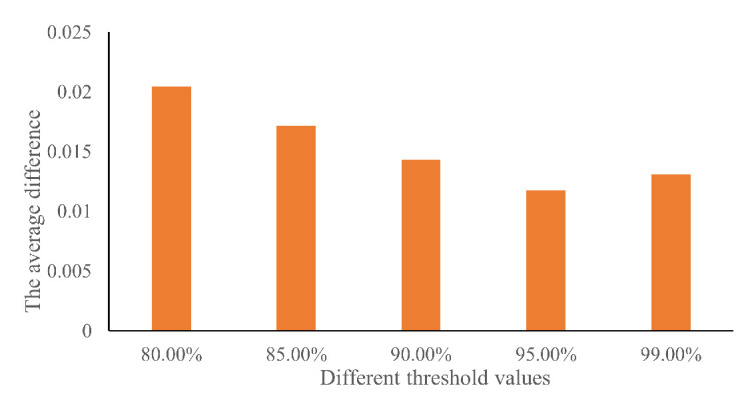
Average difference in risky behavior between training and testing datasets.

We also conducted a sensitivity analysis to evaluate the impact of the threshold value on the recognition results. We selected 80%, 85%, 90%, 95%, and 99% as threshold values to detect risky driving behavior. The absolute difference in risky behavior between the training and testing datasets with the five threshold values is shown in Table 5. The average difference is shown in Figure 13. It can be seen that the average difference decreases with the improvement in the threshold value and reaches the lowest point with the 95% threshold value. Then, the difference increases from 1.81% to 2.14% when adopting the 99% threshold value. By comparing the five threshold values, we can see that the 95% threshold value is the most suitable to recognize risky driving behavior.

To further validate the effectiveness of the proposed model, the expert scoring method was applied in this paper as the ground truth to evaluate the model. Videos of the identified risky driving behavior were extracted for the experts to evaluate the risk. Table 6 describes the accuracy of the identified risky maneuvers with expert scoring. It indicates that the average accuracy of the boxplot-based and distribution-based methods is, respectively, 90% and 85%.

The boxplot-based and distribution-based methods are both statistical methods to detect risky driving maneuvers. The boxplot-based method is a commonly used method to detect abnormal data, without the need for experienced knowledge, while the distribution-based method relies highly on the data collection amount. The threshold value based on the distribution will not be efficient if there are not enough driving samples. Therefore, the selection of the threshold extraction method depends on the data quantity.

## 5. Conclusions

A risky driving behavior recognition model is proposed based on the trajectory data extracted from videos. Three types of risky driving behavior, i.e., speed-unstable driving, serpentine driving, and risky car-following driving, are evaluated and recognized in this paper.

(1) An MOR-based risk evaluation method is proposed to establish an MOR formulation with driving features and safety surrogates for risky driving maneuvers. Two methods (distribution-based method and boxplot-based method) are applied in the MOR distribution to extract the threshold value to recognize risky maneuvers. The model is verified with a comparison of risky driving maneuvers proportion in the training and testing datasets.
(1) The proposed method can be applied to the real-time detection of risky driving behavior in video surveillance systems and provide support for the design and optimization of traffic control strategies.

Despite the merits of this study, we have to acknowledge some limitations that need to be addressed in future research. Firstly, we only concentrated on three types of risky driving behavior, which can be extended into more types with more trajectory information. Secondly, contextual factors, such as traffic flow and road type, are not taken into account. This can be addressed with more data under different contextual environments. In the future, more risky driving maneuvers can be studied in the recognition model with more features extracted from the trajectory.

## Figures and Tables

**Table 1:** Risky driving behavior classification.

	Features	Classification
Risky driving behavior classification	Based on drivers’ psychology	Aggressive driving, assertive driving
	Based on driving skill	Skilled safe driving, aggressive driving, unskilled driving, and conservative driving
	Based on traffic flow characteristics and occurrence frequency	Overspeed driving, near car-following, illegal overtaking, driving on the line, fatigue driving, frequent lane-changing
	Based on crash severity	Major accidents, minor or general accidents

**Table 2:** Sample distribution of three types of risky driving behavior.

Maneuver Type	Training Samples	Testing Samples
Speed-unstable driving	400	180
Serpentine driving	400	180
Car-following	300	100

**Table 3:** Proportion of risky driving behavior with boxplot-based method.

Dataset		Unstable Driving		Serpentine Driving		Car-Following	
		Cars	Trucks	Cars	Trucks	Cars	Trucks
Training dataset	Normal	95.25%	95.21%	96.82%	95.86%	98.55%	93.16%
	Risky	4.75%	4.79%	3.18%	4.14%	1.45%	6.84%
Testing dataset	Normal	95.98%	95.04%	96.84%	92.51%	97.98%	94.55%
	Risky	4.02%	4.96%	3.16%	7.49%	2.02%	5.45%
Absolute difference		0.73%	0.17%	0.02%	3.35%	0.57%	1.39%

**Table 4:** Proportion of risky driving behavior with distribution-based method.

Dataset		Unstable Driving		Serpentine Driving		Car-Following	
		Cars	Trucks	Cars	Trucks	Cars	Trucks
Training dataset	Normal	95.00%	95.00%	95.00%	95.00%	95.00%	95.00%
	Risky	5.00%	5.00%	5.00%	5.00%	5.00%	5.00%
Testing dataset	Normal	96.77%	94.62%	94.43%	92.33%	96.93%	93.32%
	Risky	3.23%	5.38%	5.57%	7.67%	3.07%	6.68%
Absolute difference		0.73%	1.77%	0.38%	0.57%	1.93%	1.68%

**Table 5:** Absolute difference in risky behavior between training and testing datasets with five threshold values.

Threshold Value	Unstable Driving		Serpentine Driving		Car-Following	
	Cars	Trucks	Cars	Trucks	Cars	Trucks
80%	1.27%	2.63%	0.56%	0.83%	3.53%	3.45%
85%	0.98%	2.11%	0.49%	0.78%	3.01%	2.92%
90%	0.82%	1.89%	0.41%	0.61%	2.51%	2.36%
95%	0.73%	1.77%	0.38%	0.57%	1.93%	1.68%
99%	0.75%	1.81%	0.43%	0.59%	2.35%	1.92%

**Table 6:** Accuracy of identified risky maneuvers with expert scoring.

Method	Unstable Driving		Serpentine Driving		Car-Following	
	Cars	Trucks	Cars	Trucks	Cars	Trucks
Boxplot-based method	98%	88%	92%	90%	91%	83%
Distribution-based method	92%	82%	90%	81%	87%	80%
